# Multi-UAV Cooperative Trajectory Planning Based on the Modified Cheetah Optimization Algorithm

**DOI:** 10.3390/e25091277

**Published:** 2023-08-30

**Authors:** Yuwen Fu, Shuai Yang, Bo Liu, E Xia, Duan Huang

**Affiliations:** 1 School of Automation, Central South University, Changsha 410017, China; 8213200115@csu.edu.cn (Y.F.); 212129@csu.edu.cn (E.X.); 2 School of Software Engineering, South China University of Technology, Guangzhou 510641, China; 202030484199@mail.scut.edu.cn; 3 College of Advanced Interdisciplinary Studies, National University of Defense Technology, Changsha 410073, China; liubo08@nudt.edu.cn; 4 School of Computer Science and Technology, Central South University, Changsha 410017, China

**Keywords:** autonomous trajectory planning, modified cheetah optimization algorithm, multi-unmanned aerial vehicles, adaptive search agent strategy, logistic chaotic mapping strategy

## Abstract

The capacity for autonomous functionality serves as the fundamental ability and driving force for the cross-generational upgrading of unmanned aerial vehicles (UAVs). With the disruptive transformation of artificial intelligence technology, autonomous trajectory planning based on intelligent algorithms has emerged as a key technique for enhancing UAVs’ capacity for autonomous behavior, thus holding significant research value. To address the challenges of UAV trajectory planning in complex 3D environments, this paper proposes a multi-UAV cooperative trajectory-planning method based on a Modified Cheetah Optimization (MCO) algorithm. Firstly, a spatiotemporal cooperative trajectory planning model is established, incorporating UAV-cooperative constraints and performance constraints. Evaluation criteria, including fuel consumption, altitude, and threat distribution field cost functions, are introduced. Then, based on its parent Cheetah Optimization (CO) algorithm, the MCO algorithm incorporates a logistic chaotic mapping strategy and an adaptive search agent strategy, thereby improving the home-returning mechanism. Finally, extensive simulation experiments are conducted using a considerably large test dataset containing functions with the following four characteristics: unimodal, multimodal, separable, and inseparable. Meanwhile, a strategy for dimensionality reduction searching is employed to solve the problem of autonomous trajectory planning in real-world scenarios. The results of a conducted simulation demonstrate that the MCO algorithm outperforms several other related algorithms, showcasing smaller trajectory costs, a faster convergence speed, and stabler performance. The proposed algorithm exhibits a certain degree of correctness, effectiveness, and advancement in solving the problem of multi-UAV cooperative trajectory planning.

## 1. Introduction

Unmanned aerial vehicles (UAVs) have gained increasing prominence on the battlefield, and enhancing their autonomous combat capabilities in high-threat environments has become a key research focus [1]. UAV trajectory planning is a crucial component of mission execution, as the quality of trajectory planning directly affects the survivability and mission effectiveness of UAVs [2]. The cooperative execution of missions by multiple unmanned combat aerial vehicles (UCAVs) is envisioned as the primary form of future battleground operations, making cooperative trajectory planning one of the critical technologies for enhancing UCAVs’ collaborative combat efficiency and ensuring successful mission execution [3,4]. The objective of cooperative trajectory planning is to design optimized flight paths for multiple UCAVs, within their performance limits, from the starting point to the target point/area. This problem poses a complex optimization challenge, as it requires minimizing the cost of mission execution for multiple UCAVs while meeting their cooperative requirements [5,6].

Considerable research has been conducted in the field of cooperative trajectory planning for UAVs. Presently, the research on trajectory-planning problems for single vehicles is more developed. However, in the execution of cooperative missions involving multiple UCAVs, the complexity of combat tasks often gives rise to various collaborative and performance constraints [7]. To address these issues, Cheng et al. [8] proposed a decentralized multi-UAV path-planning method specifically designed for obstacle-rich environments. This approach aims to overcome the limitations of traditional multi-UAV path-planning methods in terms of computational efficiency and scalability. In a similar vein, Liu et al. [9] analyzed the flight and cooperative constraints of UAVs and established a three-dimensional environmental model incorporating geographical information. Chen et al. [10], on the other hand, achieved kdiff multi-UAV cooperative autonomous path planning in unknown environments, considering the dynamic and partially observable nature of the environmental state. In addition, Li et al. [11] proposed a multi-drone path-planning algorithm to address challenges such as low stability, long planned paths, and low efficiency in dynamically avoiding obstacles in a three-dimensional mountainous environment. Furthermore, Wang et al. [12] introduced a multi-UAV collaborative path-planning method based on attention reinforcement learning. This method takes into account various factors, including survival probability, path length, load balancing, and endurance constraints, thereby serving as a support system for optimizing multimachine collaboration. During the execution of cooperative missions involving multiple UCAVs, multiple types of collaborative and performance constraints often arise due to the complexity of combat tasks [7]. Existing methods frequently fail to comprehensively address these constraints, resulting in ineffective trajectories that do not meet the requirements of multi-UCAV collaborative combat [13].

Because of the intricate nature of multi-constraint conditions and diverse task demands in the problem of collaborative path planning and allocation for multi-UCAV combat, researchers frequently employ meta-heuristics, such as Particle Swarm Optimization (PSO) [14,15,16], Grey Wolf Optimizer (GWO) [17,18], the Firefly Algorithm (FA) [19,20], Genetic Algorithms (GAs) [21,22], Differential Evolution (DE) algorithms [23,24], Ant Colony Optimization (ACO) algorithms [25,26], etc., to tackle the relevant computational challenges.

The Cheetah Optimizer (CO) [27] is a recently suggested optimization method proposed in 2022. By simulating three main hunting strategies commonly used by cheetahs— searching, waiting, and attacking—and introducing a strategy of returning home after leaving the prey during the hunting process, the CO algorithm aims to solve optimization problems and improve the population diversity, convergence performance, and robustness. At present, research on CO is still relatively scarce. Sait et al. [28] addressed the economic optimization challenge of plate-fin heat exchangers using the CO algorithm. The design variables were optimized using the CO algorithm, and statistical results were compared with eight well-established algorithms. Abd et al. [29] used the CO Algorithm in *Predicting Rock-Physics Parameters of Gas-Bearing Reservoirs in the Eastern Mediterranean Sea, Egypt*. Vijay et al. [30] proposed a hybrid optimization algorithm, namely, the hybrid cat cheetah optimization algorithm (the modified version of the two algorithms), that utilizes the merits of both algorithms and thus handles exploitation and exploration issues.

So far, many scholars have proposed the use of various meta-heuristic algorithms to execute the path planning of UCAVs. Xiong et al. [31] used a path-planning method employing sine–cosine particle swarm optimization (SCPSO). Kumar et al. [32] used a novel reinforcement-learning-based enhanced variable weight grey wolf optimization algorithm named RLV-GWO for Multi-UAV Path Planning. Patel et al. [33] used the firefly algorithm and a fuzzy algorithm, establishing multiple features of the individual controller, to achieve a UAV’s path and time optimization. However, the search space for the problem of multi-UCAV cooperative trajectory planning is enormous, and traditional search algorithms often face severe combinatorial explosion issues when solving this problem, resulting in excessively high planning costs [34,35]. This paper makes the following contributions:

(1) We analyze the cooperative constraints and performance constraints among multiple UCAVs. We establish a comprehensive set of trajectory-cooperative constraints that take into account spatio-temporal coordination, range, speed, angles, flight altitude, and three-dimensional threat distribution. Additionally, we propose evaluation criteria in the form of a cost function to measure the level of satisfaction of multi-type cooperative constraints among UCAV trajectories.
(2) Building upon the CO algorithm, we propose the Modified Cheetah Optimization (MCO) algorithm. This algorithm incorporates an adaptive search agent strategy, the Cheetah returning home mechanism, and the Logistic chaotic mapping strategy.
(3) The performance of the proposed approach is evaluated through testing with nine shifted CEC2005 functions and fifty test functions that cover single-peaked, multi-peaked, separable, and non-separable characteristics. The MCO algorithm is compared with other algorithms such as CO, PSO, GWO, and FA.
(4) We use the MCO algorithm in combination with a dimensionality reduction search strategy to address the problem of autonomous trajectory planning in real-world scenarios.

This paper is outlined as follows. In Section 2, we first summarize the principle of the multi-UAV system and then introduce the constraints and objectives of multi-drone trajectory planning. In Section 3, we introduce the proposed modified cheetah optimization algorithm. We provide a detailed description of the experimental setup to demonstrate the effectiveness of our method in Section 4 Finally, brief conclusions are drawn in Section 5.

## 2. Description of Cooperative Trajectory Planning Problem

Multi-UAV collaborative trajectory planning aims to optimize the trajectories of multiple UAVs to achieve collective objectives while considering various constraints and uncertainties. Let $V=\{V_{i},i=1,2,\cdots ,N_{V}\}$ be the set of UAVs assigned for task execution, $T=\{T_{i},i=1,2,\cdots ,N_{V}\}$ be the set composed of targets corresponding to each UAV, $M=\{M_{j},j=1,2,\cdots ,N_{M}\}$ be the set denoting the collection of enemy threats, and $PN=\{PN_{i},i=1,2,\cdots ,N_{V}\}$ be the set representing the collection of the number of waypoints corresponding to each UAV [36]. In this context, each individual UAV’s trajectory from the starting point to the destination point is composed of a series of waypoints. By connecting these waypoints according to certain rules between the starting and destination points, the trajectory can be obtained.

### 2.1. Collaborative Constraints

Collaborative constraints refer to ensuring that the trajectory of each UAV in a formation can successfully complete the mission as required while meeting the individual trajectory constraints [37].

#### 2.1.1. Spatial Collaborative Constraints

Spatial collaborative constraints, also known as non-collision constraints, require that the distance between any two UAVs at any given time should not be less than the minimum safe flight distance.

(1) $$\left|d_{i}-d_{j}\right|\geq d_{safe}\left(\forall i,j,i\neq j\right)$$

where $d_{i}$ and $d_{j}$ represent the position of any waypoint for the $i_{th}$ UAV and the position of any waypoint for the $j_{th}$ UAV. $d_{safe}$ represents the minimum safe flight distance between UAVs.

#### 2.1.2. Temporal Collaborative Constraints

During the collaborative execution of tasks by multiple UAVs, due to the complexity of the mission, multiple UAVs often need to arrive at their respective targets and perform tasks in a specific order [38]. This paper introduces the concept of collaborative time coordination by setting a coordinated time interval, $T_{s}$, to achieve temporal collaborative constraints among multiple UAVs.

(2) $$\left|T_{v}(i)-T_{s}\right|\leq T_{a}$$

where $T_{v}(i)$ represents the time taken for the $i_{th}$ UAV to reach its destination, $T_{s}$ represents the coordinated time interval, and $T_{a}$ represents the allowed time error value.

### 2.2. Performance Constraints

#### 2.2.1. Range Constraint and Minimum Trajectory Segment Constraint

During the execution of a mission by a drone, factors such as fuel consumption and task efficiency need to be taken into consideration. Thus, it is essential to establish the maximum range of the drone. Assuming that the maximum range $L_{max}$ of an individual UAV is:

(3) $$\sum_{j=0}^{PN_{i}+1}\left|P_{j}P_{j+1}\right|\leq L_{max}$$

where $\left|P_{j}P_{j+1}\right|$ represents the distance between the $j_{th}$ and $j+1_{th}$ waypoints in the trajectory, $PN_{i}$ represents the number of waypoints for the $i_{th}$ UAV, $P_{0}$ represents the starting point of the mission, and $P_{PN_{i}+1}$ represents the ending point of the mission.

In the constraint of the shortest trajectory segment, the maneuverability of the UAV influences the existence of a minimum straight-line distance before changing its heading. Therefore, this constraint can be expressed as:

(4) $$\left|P_{j}P_{j+1}\right|\geq L_{min}$$

where $L_{min}$ represents the shortest trajectory segment.

#### 2.2.2. Speed Constraint

During the flight process of a UAV, the flight speed needs to be maintained within a certain range. This range should take into account various factors such as local weather conditions, wind speed, the number of restricted flight zones, and more. It is essential to control the speed of the unmanned aircraft within this range.

(5) $$v_{min}\leq v_{i}\leq v_{max}$$

where i represents the UAV number and $v_{min}$ and $v_{max}$ represent the minimum and maximum speeds, respectively.

#### 2.2.3. Angle Constraint

The angle constraint includes two parts: the yaw angle constraint and the pitch angle constraint. The yaw angle constraint refers to the limitation on the turning angle of the UAV during flight between two consecutive waypoints. Similarly, the pitch angle constraint specifies that the vertical angle of the UAV can only vary within a certain range between two consecutive waypoints. The yaw and pitch angles of the UAV are affected by its thrust and maneuverability. If the angles exceed this range, there is a risk of crashing. The UAV angle constraint is as follows:

(6) $$\theta_{min}\leq \theta \leq \theta_{max}$$

(7) $$\varphi_{min}\leq \varphi \leq \varphi_{max}$$

where θ represents the yaw angle, $\theta_{min}$ and $\theta_{max}$ represent the maximum and minimum allowed values of the yaw angle, respectively, φ represents the pitch angle, and $\varphi_{min}$ and $\varphi_{max}$ represent the maximum and minimum allowed values of the pitch angle, respectively.

#### 2.2.4. Flight Altitude Constraint

In the spatial planning of UAVs, the flight altitude should not be lower than the minimum flight altitude to prevent the risk of crashing into the ground due to excessively low altitude. Additionally, the waypoints of the UAV trajectory should be within the planned airspace. Therefore, the flight altitude should be maintained below the maximum flight altitude. The flight altitude constraint is expressed as follows:

(8) $$h_{min}\leq h_{j}^{i}\leq h_{max}$$

where $h_{min}$ denotes the minimum value of the flight altitude, $h_{max}$ represents the maximum value of the flight altitude, and $h_{j}^{i}$ denotes the flight altitude of the *i*th waypoint of an individual unmanned aerial vehicle (UAV).

#### 2.2.5. Three-Dimensional Threats Spatial Distribution Constraint

In the airspace of unmanned aerial vehicle (UAV) operations, there are often three-dimensional threat distributions, such as radar systems, missiles, and anti-aircraft artillery. During the collaborative mission execution of multiple UAVs, it is crucial to avoid entering these threat areas. The model of three-dimensional threat distribution can be described as:

(9) $$M_{j}=\left\{M_{jx},M_{jy},M_{jz},M_{jr}\right\}$$

where $\left\{M_{jx},M_{jy},M_{jz}\right\}$ represents the central coordinates of the threat model $M_{j}$ and $M_{jr}$ represents the effective range of influence.

### 2.3. Cost Functions

The evaluation of the quality of UAV trajectories is composed of various indicators. The evaluation indicators of the trajectory cost mainly include fuel cost, altitude cost, and threat cost. These cost functions serve as standards for assessing the superiority or inferiority of trajectories. Therefore, the cost function can be defined as:

(10) $$\min f=\alpha_{1}*f_{1}+\alpha_{2}*f_{2}+\alpha_{3}*f_{3}+\alpha_{4}*f_{4}+\alpha_{5}*f_{5}$$

where $f_{1}$,$f_{2}$,$f_{3}$,$f_{4}$, and $f_{5}$ represent the track cost, altitude cost, threat cost, spatial coordination cost, and temporal coordination cost, respectively. The respective weight parameters for these factors are denoted as $\alpha_{1}$,$\alpha_{2}$,$\alpha_{3}$, and $\alpha_{4}$.

#### 2.3.1. Trajectory Length Cost

Fuel consumption is one of the important evaluation indicators for mission allocation. The duration of a drone’s flight indirectly reflects the amount of fuel consumed. The expression for calculating the cost of the trajectory length is as follows:

(11) $$f_{1}=\sum_{i=1}^{N_{V}}\sum_{j=0}^{PN_{i}+1}\left|P_{j}^{i}P_{j+1}^{i}\right|$$

where $\left|P_{j}^{i}P_{j+1}^{i}\right|$ refers to the length of the flight segment between the $j_{th}$ and $j+1_{th}$ waypoints for the $i_{th}$ unmanned aerial vehicle.

#### 2.3.2. Height Cost

When the flight altitude of unmanned aerial vehicles exceeds the designated height range, a height cost is incurred, which can be expressed as follows:

(12) $$f_{h_{i}}=\left\{\begin{array}{ll} \gamma_{1}*\left(h_{j}^{i}-h_{max}\right), & h_{j}^{i}>h_{max}\\ 0, & h_{min}<h_{j}^{i}<h_{max}\\ \gamma_{2}*\left(h_{min}-h_{j}^{i}\right), & h_{j}^{i}<h_{\min} \end{array}\right.$$

where $\gamma_{1}$ and $\gamma_{2}$ are proportionality coefficients. Therefore, the expression for the height cost is as follows:

(13) $$f_{3}=\sum_{i=1}^{N_{V}}f_{h_{i}}$$

#### 2.3.3. Threat Zone Cost

Due to the presence of various spatial threats such as radar, missiles, and anti-aircraft artillery in UAV flight space, the cost of threats includes radar, missiles, anti-aircraft artillery, and atmospheric threats. This article defines the threat cost for different threats as follows:

The detection probability of radar for unmanned aerial vehicles (UAVs) can be approximately represented as

(14) $$f_{t}=\left\{\begin{array}{cc} 0, & V_{x}-Or_{M}>R_{M}\\ 1/{\left(\left|V_{x}-Or_{M}\right|\right)}^{4}, & V_{x}-Or_{M}\leq R_{M} \end{array}\right.$$

where $\left|V_{x}-Or_{M}\right|$ represents the distance of the UAV from the radar center and $R_{M}$ is the threat radius of the radar. 

The detection probability of other threats such as missiles and anti-aircraft artillery for the UAV can be approximately represented as

(15) $$f_{t}=\left\{\begin{array}{cc} 0, & V_{x}-Or_{O}>R_{O}\\ 1/\left|V_{x}-Or_{0}\right|, & V_{x}-Or_{O}\leq R_{O} \end{array}\right.$$

where $\left|V_{x}-Or_{0}\right|$ represents the distance of the UAV from the threat center and $R_{O}$ is the threat radius. 

The expression for the threat cost is as follows:

(16) $$f_{4}=\sum f_{t}$$

#### 2.3.4. Spatial Collaboration Cost

After obtaining the trajectory planning results for multiple UAVs, collision checking is performed on the trajectories. Let $f_{c}$ be the total number of collisions. The spatial collaboration cost is expressed as follows:

(17) $$f_{5}=\delta *\sum f_{c}$$

where δ is the proportionality coefficient.

#### 2.3.5. Temporal Collaboration Cost

Assuming that UAVs maintain a constant speed during the flight process, the temporal collaboration cost can be computed by calculating the time taken for UAVs to reach their respective targets. The expression for the temporal collaboration cost is as follows:

(18) $$T_{v(i)}=L_{i}/v_{i}$$

(19) $$f_{k}=\left\{\begin{array}{cc} 0, & \left|T_{v(i)}-T_{s}\right|\leq T_{a}\\ \sigma *\left|T_{v(i)}-T_{s}\right|, & \left|T_{v(i)}-T_{s}\right|>T_{a} \end{array}\right.$$

where σ is the proportionality coefficient, $L_{i}$ is the total distance traveled by the $i_{th}$ UAV, and $T_{v(i)}$ is the time taken by it.

(20) $$f_{6}=\sum f_{k}$$

## 3. Solving Multi-UCAV Trajectory Planning Problems by MCO

### 3.1. Cheetah Optimization (CO) Algorithm

The cheetah optimization (CO) algorithm is a novel heuristic intelligent optimization algorithm based on the hunting strategies of cheetahs in nature. By simulating three main hunting strategies commonly used by cheetahs, searching, waiting, and attacking, and introducing a strategy of returning home after leaving the prey during the hunting process, CO aims to solve optimization problems and improve the algorithm’s population diversity, convergence performance, and robustness [27].

One of the main hunting strategies employed by the CO algorithm is the searching strategy. The following equation presents the random search equation that updates the new position of the cheetah based on its current position within each permutation:

(21) $$X_{i,j}^{t+1}=X_{i,j}^{t}+{\hat{\gamma }}_{i,j}^{-1}\cdot \alpha_{i,j}^{t}$$

where $X_{i,j}^{t+1}$ and $X_{i,j}^{t}$ are the next and current positions of the $i_{th}$ cheetah in the permutation j, respectively. The index t denotes the current hunting time and T represents the maximum duration of the hunting time. ${\hat{\gamma }}_{i,j}^{-1}$ and $\alpha_{i,j}^{t}$ are the randomization parameter and step length of the $i_{th}$ cheetah in the permutation j, respectively. The randomization parameter ${\hat{\gamma }}_{i,j}$ is a normally distributed random number generated from a standard normal distribution. In most cases, the step length $\alpha_{i,j}^{t}>0$ can be set to a small value 0.001×t/T, making the cheetahs slow-walking searchers.

In the waiting strategy, the cheetah remains stationary and waits for the prey to approach. This behavior can be modeled as follows:

(22) $$X_{i,j}^{t+1}=X_{i,j}^{t}$$

where $X_{i,j}^{t+1}$ and $X_{i,j}^{t}$ are the updated and current positions of the $i_{th}$ cheetah in the permutation j, respectively. This strategy requires the CO algorithm to selectively change the positions of cheetahs within each group to increase the success rate of hunting (finding better solutions). It helps the algorithm to avoid premature convergence.

The attacking strategy of cheetahs can be defined mathematically as:

(23) $$X_{i,j}^{t+1}=X_{B,j}^{t}+{\overset{⌣}{\gamma }}_{i,j}\cdot \beta_{i,j}^{t}$$

where $X_{B,j}^{t}$ is the current position of the prey in the permutation j, ${\overset{⌣}{\gamma }}_{i,j}$ and $\beta_{i,j}^{t}$ are the turning factor and interaction factor associated with the $i_{th}$ cheetah in the permutation j. The turning factor $\beta_{i,j}^{t}$ reflects the interaction between cheetahs or between cheetahs and the leader in the capturing pattern. Mathematically, this factor can be defined as the difference between the positions of nearby cheetahs $X_{k,j}^{t}(k\neq i)$ and the position $X_{i,j}^{t}$ of the $i_{th}$ cheetah. The turning factor ${\overset{⌣}{\gamma }}_{i,j}$ is a random number equal to ${\left|r_{i,j}\right|}^{\exp(r_{i,j}/2)}\sin(2\pi r_{i,j})$. $r_{i,j}$ is a random number generated from a standard normal distribution. This factor reflects the sharp turns made by cheetahs in the capturing pattern.

Due to the limitation of energy, the hunting time of each group of cheetahs is finite. Therefore, if a group fails to succeed within a certain hunting time, the current prey is left behind, and the group returns to its activity range for rest before starting another hunting session. In fact, if the energy of the cheetahs (modeled by hunting time) decreases while the position of the leader remains unchanged, a group of cheetahs will return home. At this point, the position of the leader is also updated. The result of this strategy is to avoid becoming trapped in local optima.

### 3.2. Modified Cheetah Optimization (MCO) Algorithm

#### 3.2.1. Improved Population Position Updating Method

The quality of the initial population significantly affects the accuracy and convergence speed of an algorithm, and an initial population with good diversity can greatly improve the performance of the algorithm [39]. However, in the CO algorithm, a random method is typically used to generate the initial population when solving optimization problems. This may result in an uneven distribution of the initial population and poor diversity. Additionally, the strategy of cheetahs returning to their initial home is a key factor in the optimization process of the CO algorithm. Therefore, a uniformly distributed initial population can effectively improve the efficiency of the solution and lay the foundation for diversity in the algorithm’s global search. In this paper, we utilize logistic chaotic mapping [40] to initialize the population. Compared to other chaotic mappings, logistic chaotic mapping has demonstrated excellent optimization performance and convergence theory optima for both unimodal and multimodal functions. It exhibits strong convergence and the ability to escape local optima. Thus, logistic chaotic mapping is employed for population initialization in this study. The expression of the logistic chaotic mapping is as follows:

(24) $$x_{k+1}=\lambda *x_{k}(1-x_{k}),\lambda \in (0,4)$$

**Figure 1:**
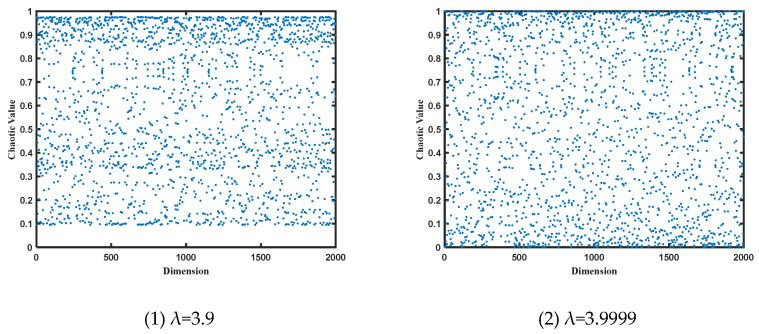
The relationship between logistic chaotic mapping and iteration times under different values of λ.

In Figure 1, it can be observed that as the parameter λ increases, the value of x tends to be uniformly distributed in the interval [0, 1]. By applying logistic chaotic mapping to the CO algorithm, the uniformity of the initial solution’s distribution is enhanced, leading to improved optimization efficiency and traversal uniformity. This approach improves the collective search capability and, to some extent, overcomes the limitations of reduced population diversity, susceptibility to local optima, and decreased search accuracy typically encountered by swarm intelligence algorithms when approaching the optimal solution.

After initialization of the population according to Equation (25), it needs to be mapped to the solution space as follows:

(25) X=lb+(ub−lb)·x

where x is the logistic chaotic sequence generated through Equation (25), and X is the initial population generated through chaotic mapping.

#### 3.2.2. Adaptive Search Agent Strategy

In each iteration, a subset of individuals participates in the evolution process, which is referred to as the search agent. In the CO algorithm, the number of search agents remains fixed throughout the entire iteration process. When the number of search agents is small, the algorithm is prone to becoming trapped in local optima and has poor global search ability. On the other hand, when the number of search agents is large, the convergence speed of the algorithm becomes slow. To achieve a better balance between the global and local search abilities, this paper proposes a new formula for calculating the number of search agents which decreases non-linearly with iteration count. The specific expression is as follows:

(26) $$m=(m_{max}-m_{min})\cos\left(\frac{\pi \cdot it}{2MaxIt}\right)$$

where $m_{max}$ is the maximum value for the convergence factor, which is set to n in this paper, $m_{min}$ represents the minimum value of the convergence factor and is set to 2, it is the iteration count, and MaxIt is the maximum number of iterations.

**Figure 2:**
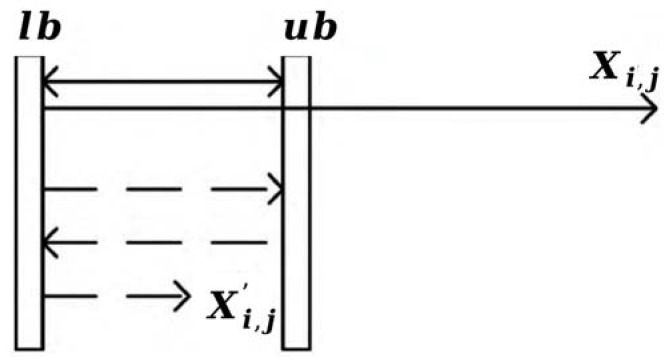
Illustration of the dual-mirror-reflection theory for the MCO algorithm which can effectively address the issue of uneven distribution during boundary handling.

During the search process, the CO algorithm utilizes the dual-mirror-reflection theory for boundary optimization [41]. When individuals exceed the boundary, the CO algorithm assigns them the values of the upper or lower boundary directly. This leads to the clustering of solutions at the boundaries, resulting in sparse distribution in other regions. The uneven distribution of individuals can directly impact the performance of the algorithm. In this paper, the dual-mirror-reflection boundary handling approach is employed, treating the upper and lower boundaries ub and lb as two mirrors and the individuals $X_{i,j}$ as propagating beams. The size of the beams, denoted as $X_{i,j}$, represents the intensity of the light. After multiple reflections, the beams eventually vanish within the boundaries $X_{i,j}{}^{\prime }$ due to medium losses, as illustrated in Figure 2. Thus, the projection $X_{i,j}{}^{\prime }$ of $X_{i,j}$ within the boundaries serves as a solution representation. This approach effectively solves the issue of uneven distribution caused by boundary handling.

The formula for the dual-mirror-reflection boundary handling approach is as follows:

(27) $$X_{i,j}{}^{\prime }=\left\{\begin{array}{ll} ub-\mathrm{mod}(X_{i,j}-ub,ub-lb), & X_{i,j}>ub\\ lb+\mathrm{mod}(lb-X_{i,j},ub-lb), & X_{i,j}<lb \end{array}\right.$$

#### 3.2.3. Strategy of Cheetah Returning to Home after Leaving Prey

In the later stages of the CO algorithm evolution, the probability of cheetahs leaving their prey and returning to their home increases, and the leader’s position is updated. Therefore, an efficient method of updating the leader’s position is particularly important. In contrast to the random updating method employed in the original algorithm, this study introduces the Cauchy mutation operator [42] to update the leader’s position in order to maintain a balance between population diversity and algorithm convergence during the evolution process. This method effectively enhances the algorithm’s ability to escape local optima and avoid premature convergence. The formula is as follows:

(28) $$X_{BestSol}=X_{BestSol}+\eta \times C(0,1)$$

(29) $$\eta =e^{-\lambda \cdot \frac{it}{MaxIt}}$$

where $X_{BestSol}$ represents the global optimum solution at generation it; η is the mutation weight; C(0,1) is the standard Cauchy random distribution at generation it=1; and λ is an adjustment parameter with a range of [30,100].

After some cheetahs return to their home, the cheetah population will engage in a reverse search in order to locate their prey more quickly. The reverse search strategy [43] is based on the current solution and employs a reverse learning mechanism to find the corresponding reverse solution. The better solutions are then evaluated and compared for preservation. According to probability theory, there is a 50% probability that the current solution is further away from the optimal solution compared to its reverse solution. Therefore, if the cheetah simultaneously searches the current solution and the reverse solution during the search process, selecting the better solution as the predicted solution will greatly improve the efficiency of the cheetah in capturing its prey. The expression for the reverse search strategy is as follows:

(30) $${\hat{X}}_{i}=ub+lb-X_{i}$$

(31) $$X_{i}^{new}=\left\{\begin{array}{ll} {\hat{X}}_{i}, & fit({\hat{X}}_{i})<fit(X_{i})\\ X_{i}, & fit({\hat{X}}_{i})>fit(X_{i}) \end{array}\right.$$

where $X_{i}$ represents the current solution of the cheetah population, ${\hat{X}}_{i}$ is the reverse solution of the cheetah population, $fit(X_{i})$ represents the fitness value of the current solution of the cheetah population, $fit({\hat{X}}_{i})$ represents the fitness value of the reverse solution of the cheetah population, and $X_{i}^{new}$ represents the updated cheetah position.

Based on the above considerations, the pseudocode for the MCO algorithm is as follows (Algorithm 1):
**Algorithm 1:** The MCO Algorithm1:Define the problem data, dimension (D), and the initial population size (n)2:Generate the initial population of cheetahs $X_{i}(i=1,2,\cdots ,n)$ and evaluate the fitness of each cheetah3:Initialize the population’s home, leader and prey solutions, using logistic chaos theory4:t←05:it←16:MaxIt← desired maximum number of iterations7:$T\leftarrow 60\times \left\lceil D/10\right\rceil$8:**while** it≤MaxIt do 
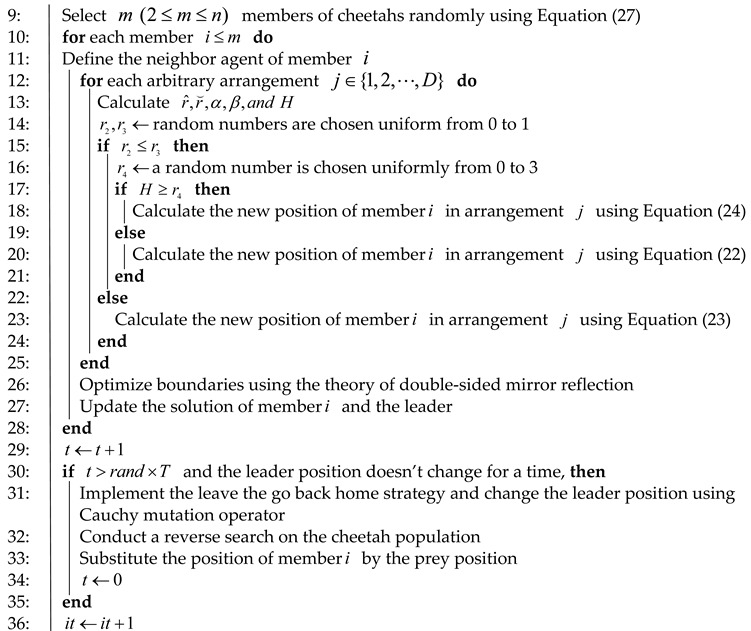
37: Update the prey (global best) solution
38:**end**

### 3.3. Encoding Strategy

**Figure 3:**
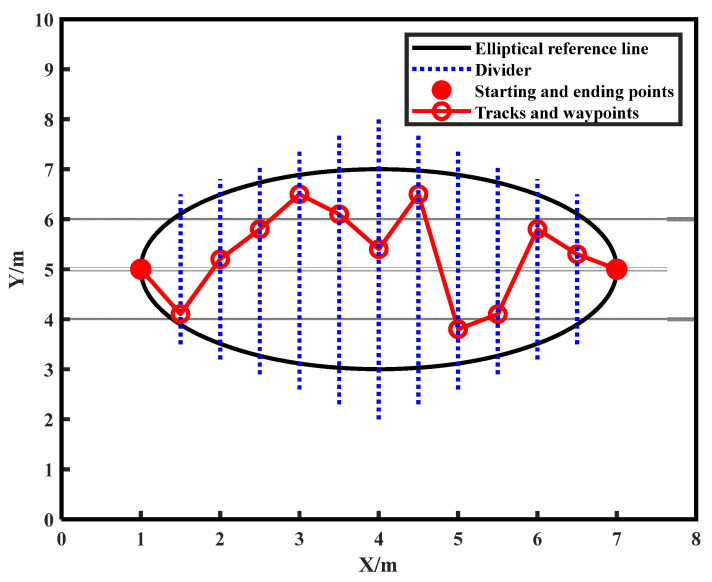
Mathematical model illustration of the UAV offline trajectory planning based on a dimensionality reduction search. This model employs elliptical partition lines to depict the trajectory of the UAV.

In establishing a three-dimensional UAV trajectory planning model, if the problem dimension is set too high, intelligent optimization algorithms exhibit instability, especially in complex situations where the algorithms struggle to converge within a reasonable time [44]. Therefore, this study adopts a dimension-reduction search strategy. The mathematical model for offline UAV trajectory planning is represented as follows (Figure 3):

Projecting the start and end points onto the plane xoy, we establish an ellipse equation using the start and end points as the endpoints of the major axis. Connecting the start and end points forms the major axis of the ellipse. Based on the number of individual UAV trajectory points, $PN_{i}$, we generate $PN_{i}$ equally spaced division lines that divide the ellipse’s major axis. The intersection points between these division lines and the ellipse are used as the reference points for each trajectory point during the trajectory planning process. It is essential to ensure that the coordinates of each individual trajectory point lie on the division lines x and y, and vary within the range of the reference points.

The encoding rules are as follows: assuming we have $N_{V}$ UAVs and their sets of trajectory points $PN=\{PN_{i},i=1,2,\cdots ,N_{V}\}$, in order to determine the collaborative UAV trajectory planning scheme, the search space for a single cheetah $X_{i}$ is represented by the sequence $\left\{x_{i,1},x_{i,2},x_{i,3},\cdots ,x_{i,j},x_{i,j+1},x_{i,j+2},\cdots ,x_{i,j+N_{V}}\right\}$. Here, $j=\sum_{i}^{N_{V}}PN_{i}$ represents the total number j of UAV trajectory points, $x_{i,1}\sim x_{i,j}$ represents the coordinates of all UAV trajectory points x, and $x_{i,j+1}\sim x_{i,j+N_{V}}$ represents the speed of each UAV in the trajectory plan. According to the mathematical model, for a single UAV, given the coordinates of the trajectory point x, we can obtain the corresponding coordinates of the point y. The coordinates z, on the other hand, are generated based on a uniform distribution, considering the height difference between the start and end points and the number of individual UAV trajectory points.

## 4. Experimental Results and Applications

To substantiate the efficacy of the proposed MCO algorithm, we conducted preliminary tests on this method using publicly accessible benchmark functions. Subsequently, we implemented the MCO algorithm to address multi-UCAV trajectory-planning challenges. The experiments were conducted using MATLAB_ R2018a and performed on a computer equipped with a 1.1 GHz dual-core Intel Core i3 processor, 8GB of memory, and an Intel Iris Plus Graphics 1536 MB graphics card. The experiment utilized macOS Big Sur 11.7.6 as the operating system.

### 4.1. Test of Public Benchmark Functions

In this section, we tested nine CEC2005 functions with shifts [45] and fifty test functions (F50) [46]. The set of 50 test functions is considerably large, covering functions with four characteristics: unimodal, multimodal, separable, and inseparable. Unimodal functions have only one local extremum, while multimodal functions have multiple local extrema. The multimodal nature of these functions makes it easy for algorithms to obtain local optima. The separability characteristic implies that the variables of a function can be decomposed into the product of functions of each variable independently, while the inseparability characteristic indicates otherwise, as the variables are interrelated. This inseparability often makes it challenging to find the global optimum. These characteristics were utilized to evaluate the performance of the proposed modified optimization algorithm and compare the results with the CO, PSO [14], GWO [17], and FA [19] algorithms. Among these algorithms, the parameters of the search agents are as follows: in the CO algorithm, m=2; in the PSO algorithm, c1 = c2 = 2.0, w=0.9; in the FA algorithm, beta0=2.0, gamma = 1.0, alpha = 0.2, and alpha damp = 0.98. We analyzed the Min(Minimum), Mean and Standard Deviation (SD) of the fitness values in all experiments. Additionally, we have highlighted the results of the algorithm presenting the best performance. We used the Friedman test values that can reflect the difference between the proposed MCO algorithm and other algorithms.

**Figure 4:**
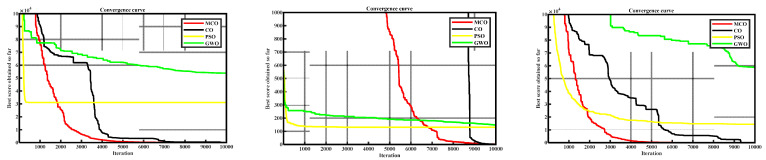

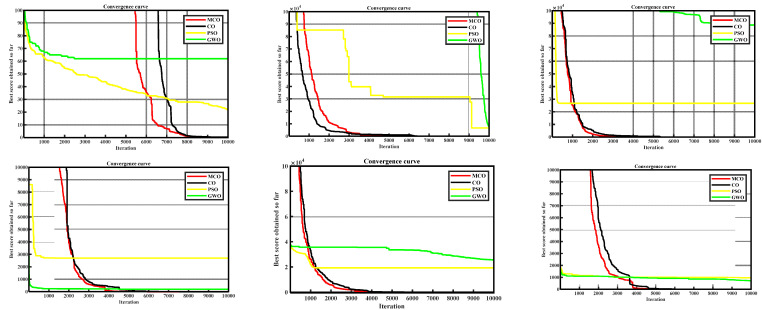
Comparative convergence curves from tests on nine CEC2005 functions with shifts.

Based on the data presented in Table 1, Table 2, Table 3 and Table 4, and the convergence curves in Figure 4, the MCO algorithm exhibited exceptional solving capability and strong stability in terms of comparing the optimal extremes, means, and standard deviations. In general, the MCO algorithm was proven effective in enhancing CO’s development, exploration, and stability capacities. Furthermore, in the majority of cases, the MCO algorithm’s convergence speed and accuracy also surpassed other algorithms.

### 4.2. Test of Multi-UCAV Trajectory Planning Problems

**Figure 5:**
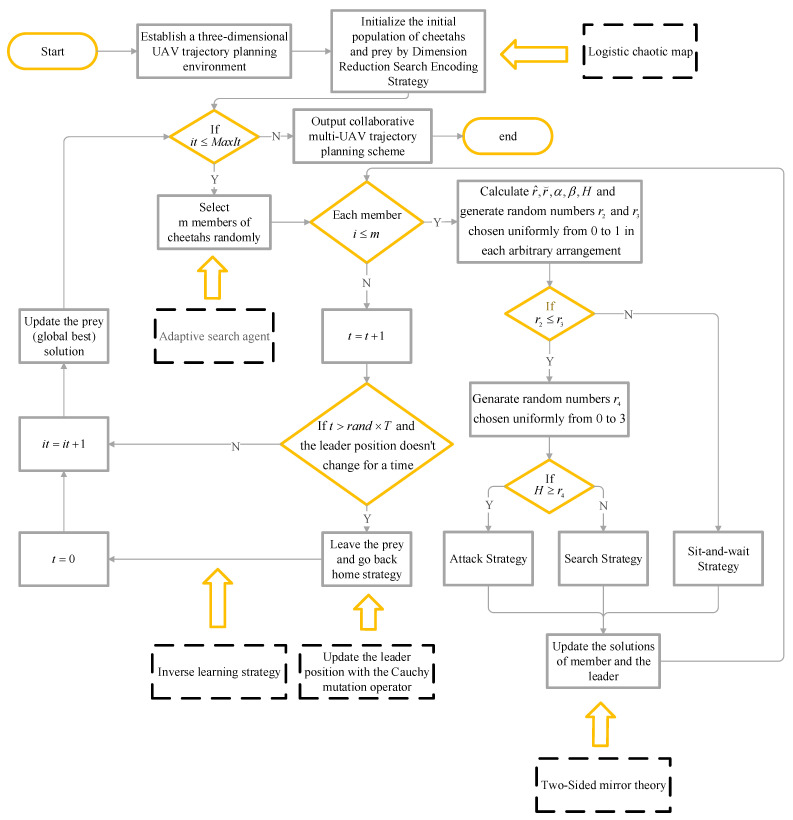
Flow chart of the experimental steps for multi-UAV cooperative path planning using the MCO algorithm.

The experimental framework for UAV path planning based on the MCO algorithm proposed in this study is illustrated in Figure 5.

In this section, the proposed MCO algorithm is evaluated in four distinct scenarios, namely, PSO, GWO, FA, MCO, and CO. Among these algorithms, the parameters of the search agents are as follows: in the CO algorithm, m=2; in the PSO algorithm, c1 = c2 = 2.0, w = 0.9; in FA algorithm, beta0 = 2.0, gamma = 1.0, alpha = 0.2, and alpha damp = 0.98. The starting and target positions of the UAVs, the stereoscopic threat distribution field position and the effective operating distance are presented in Table 5. The allowable maximum and minimum values for the yaw angle ($\theta_{min}$ and $\theta_{max}$) and pitch angle ($\varphi_{min}$ and $\varphi_{max}$) were set to −60 and 60 and −45 and 45, respectively. The values of the weight coefficients $\alpha_{1}$,$\alpha_{2}$,$\alpha_{3}$,$\alpha_{4}$, and $\alpha_{5}$ were set as 0.85, 0.45, 0.9, 0.7, and 0.7, respectively.

**Figure 6:**
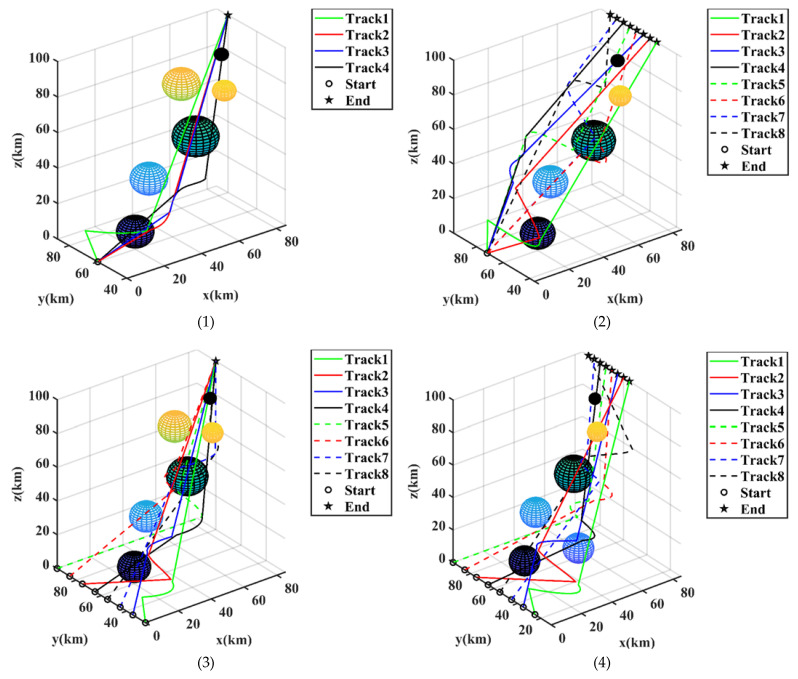
Simulation result figures for UAV path planning in the four scenarios. The planned trajectories were provided based on the outcomes shown in the figures.

The MCO algorithm was employed to perform path planning for UAVs in four different scenarios, with a population size of 50 and a total of 2000 iterations. In Scenario 1, all the UAVs had the same initial positions and corresponding target coordinates. In Scenario 2, all the UAVs had the same initial positions, but their target coordinates were different. In Scenario 3, the initial positions of all the UAVs were diverse, while their target coordinates remained the same. In Scenario 4, both the initial positions and target coordinates of all the UAVs were distinct. The result of multi-drone trajectory cooperative planning based on the multi-objective optimization (MCO) algorithm is shown in Figure 6.

**Figure 7:**
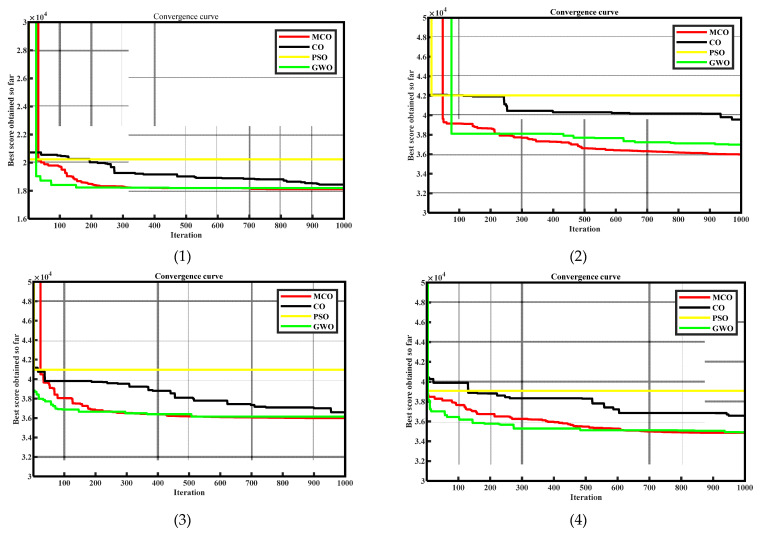
Comparative convergence curves of four algorithms obtained by conducting 50 simulations in four different scenarios.

To further compare the effectiveness of the four algorithms, namely MCO, CO, PSO, and GWO, they were individually run 50 times in each of the four scenarios. In all cases, the population size was set to 50, and the number of iterations was set to 1000. The convergence comparison curves are shown in Figure 7. The optimization extreme values, means, and standard deviations obtained from the 50 simulations are compared in Table 6. It is evident that the MCO algorithm exhibited a significantly faster convergence speed and better optimization accuracy compared to the CO algorithm. Moreover, compared to the other comparative algorithms, MCO demonstrated a superior overall optimization performance in terms of both speed and stability.

## 5. Conclusions

This study proposes a new algorithm named MCO that integrates a mixed strategy consisting of three improved techniques for solving the problem of multi-UAV collaborative trajectory planning in a three-dimensional environment. By introducing logistic chaotic mapping, the algorithm improves the initialization of population positions. By employing an adaptive search agent strategy, the algorithm effectively balances global and local search capabilities and utilizes the bidirectional mirror-reflection theory for boundary optimization to effectively address the problem of uneven distribution when handling boundaries. By introducing the Cauchy mutation operator to update leader positions, the diversity of the population was increased, effectively enhancing the algorithm’s ability to escape local optima. The proposed MCO algorithm has been tested on 59 benchmark functions, and we conducted a Friedman test on the result. The experimental results show that the performance of the MCO algorithm is significantly better than that of the other algorithms, showing advantages in terms of optimizing extremum values, means, and stability. Finally, the proposed MCO algorithm was applied to the problem of multi-UAV collaborative trajectory planning along with the CO, PSO, and GWO algorithms. Simulation experiments demonstrated that the MCO achieved more stable application effects and higher-quality planned paths, exhibiting improvements in both speed and stability. In future work, techniques such as crossover operators can be introduced to further enhance the performance of the MCO algorithm. Additionally, consideration can be given to collision risk costs and communication costs under signal denial conditions in relation to the problem of multi-UAV collaborative trajectory planning.

## Figures and Tables

**Table 1:** Test on nine CEC2005 functions with shifts. The population size for all algorithms was set to six in the nine functions, with a maximum number of iterations of D × 100, and each algorithm was run for 50 iterations.

Functions	Properties	MCO	CO	PSO	GWO
f1	Min	**2.19 × 10^−3^**	2.71 × 10^−3^	7.82 × 10^3^	3.84 × 10^4^
	Mean	**9.11 × 10^−2^**	2.85 × 10^−1^	3.15 × 10^4^	6.51 × 10^4^
	SD	**1.63 × 10^−1^**	9.90 × 10^−1^	1.32 × 10^4^	1.32 × 10^4^
f2	Min	**2.25 × 10^−3^**	3.88 × 10^−3^	9.70 × 10^1^	1.39 × 10^2^
	Mean	**5.02 × 10^−2^**	9.67 × 10^−2^	1.47 × 10^2^	1.68 × 10^2^
	SD	7.30 × 10^−2^	**1.97 × 10^−2^**	2.85 × 10^1^	1.44 × 10^1^
f3	Min	5.31 × 10^−3^	**2.07 × 10^−3^**	3.87 × 10^3^	1.17 × 10^5^
	Mean	**8.91 × 10^−2^**	1.14 × 10^−1^	3.75 × 10^4^	1.70 × 10^5^
	SD	**1.82 × 10^−1^**	1.93 × 10^−1^	2.51 × 10^4^	1.56 × 10^5^
f4	Min	4.17 × 10^−3^	**2.72 × 10^−3^**	1.94 × 10^1^	6.20 × 10^1^
	Mean	**1.57 × 10^−1^**	1.58 × 10^−1^	2.84 × 10^1^	6.51 × 10^1^
	SD	**2.81 × 10^−1^**	3.59 × 10^−1^	4.20 × 10^0^	1.65 × 10^0^
f5	Min	**4.21 × 10^−3^**	5.56 × 10^−3^	1.38 × 10^6^	1.07 × 10^8^
	Mean	**1.27 × 10^−1^**	3.80 × 10^−1^	2.34 × 10^7^	2.33 × 10^8^
	SD	**2.59 × 10^−1^**	1.34 × 10^0^	2.07 × 10^7^	6.12 × 10^7^
f6	Min	**3.83 × 10^−3^**	6.07 × 10^−3^	7.44 × 10^3^	3.64 × 10^4^
	Mean	**1.58 × 10^−1^**	4.71 × 10^−1^	3.17 × 10^4^	6.58 × 10^4^
	SD	**4.09 × 10^−1^**	1.50 × 10^0^	1.57 × 10^4^	1.42 × 10^4^
f7	Min	**2.04 × 10^−3^**	6.06 × 10^−3^	1.30 × 10^3^	8.85 × 10^1^
	Mean	**1.01 × 10^−1^**	1.27 × 10^−1^	3.47 × 10^3^	2.15 × 10^2^
	SD	2.41 × 10^−1^	**2.26 × 10^−1^**	7.59 × 10^2^	7.10 × 10^1^
f8	Min	3.19 × 10^−3^	**1.51 × 10^−3^**	1.64 × 10^4^	2.27 × 10^4^
	Mean	**1.57 × 10^−1^**	2.77 × 10^−1^	1.99 × 10^4^	2.64 × 10^4^
	SD	**3.42 × 10^−1^**	9.00 × 10^−1^	1.83 × 10^3^	1.96 × 10^3^
f9	Min	**1.65 × 10^−3^**	5.69 × 10^−3^	9.70 × 10^2^	6.06 × 10^2^
	Mean	**1.25 × 10^−1^**	8.70 × 10^−1^	1.10 × 10^3^	7.31 × 10^2^
	SD	**2.91 × 10^−1^**	4.95 × 10^0^	6.30 × 10^1^	6.70 × 10^1^

**Table 2:** Friedman test statistical results of four algorithms on 9 CEC2005 functions.

Source	SS	df	MS	Chi-sq	Prob > Chi-sq
Columns	115.204	3	38.4012	69.38	5.79677 × 10^−15^
Error	19.296	78	0.2474		
Total	134.5	107			

**Table 3:** Test on 50 test functions F50. The population size for all algorithms was set to six, with a maximum number of iterations of D × 100, and each algorithm was run for 100 iterations.

Functions	Properties	MCO	CO	PSO	FA
f1	Min	**−5.00 × 10^0^**	−5.00 × 10^0^	−5.00 × 10^0^	−4.00 × 10^0^
	Mean	**−4.34 × 10^0^**	−3.60 × 10^0^	−4.34 × 10^0^	2.20 × 10^−1^
	SD	**7.81 × 10^−1^**	1.03 × 10^0^	2.64 × 10^0^	2.74 × 10^0^
f2	Min	**0.00 × 10^0^**	0.00 × 10^0^	1.10 × 10^1^	6.50 × 10^1^
	Mean	**7.79 × 10^0^**	1.67 × 10^1^	1.50 × 10^1^	1.22 × 10^3^
	SD	1.42 × 10^1^	5.54 × 10^1^	**1.52 × 10^0^**	2.16 × 10^3^
f3	Min	1.54 × 10^−7^	**5.43 × 10^−8^**	1.07 × 10^1^	5.23 × 10^1^
	Mean	**1.24 × 10^−5^**	2.17 × 10^−5^	1.39 × 10^1^	2.62 × 10^2^
	SD	**3.54 × 10^−5^**	5.98 × 10^−5^	1.34 × 10^0^	1.76 × 10^2^
f4	Min	**3.92 × 10^−9^**	1.77 × 10^−8^	1.28 × 10^2^	3.83 × 10^2^
	Mean	**1.94 × 10^−6^**	2.76 × 10^−6^	1.94 × 10^2^	1.73 × 10^3^
	SD	**5.87 × 10^−6^**	1.12 × 10^−5^	2.84 × 10^1^	1.23 × 10^3^
f5	Min	**3.40 × 10^−2^**	4.71 × 10^−2^	4.28 × 10^1^	2.40 × 10^1^
	Mean	**1.74 × 10^−1^**	2.10 × 10^−1^	9.61 × 10^1^	2.40 × 10^1^
	SD	**8.61 × 10^−2^**	1.02 × 10^−1^	1.85 × 10^1^	2.71 × 10^1^
f6	Min	**2.38 × 10^−15^**	1.16 × 10^−14^	8.92 × 10^−6^	5.89 × 10^−7^
	Mean	5.04 × 10^−2^	1.24 × 10^−1^	1.07 × 10^−1^	**4.01 × 10^−2^**
	SD	**2.01 × 10^−1^**	2.86 × 10^−1^	2.67 × 10^−1^	2.00 × 10^−1^
f7	Min	**−1.00 × 10^0^**	−1.00 × 10^0^	−1.00 × 10^0^	−1.00 × 10^0^
	Mean	−8.47 × 10^−1^	−7.42 × 10^−1^	**−9.96 × 10^−1^**	−3.40 × 10^−1^
	SD	3.35 × 10^−1^	4.09 × 10^−1^	**5.20 × 10^−3^**	4.78 × 10^−1^
f8	Min	1.84 × 10^−9^	**2.65 × 10^−12^**	3.29 × 10^−8^	3.05 × 10^−8^
	Mean	1.08 × 10^−3^	1.29 × 10^−3^	**5.02 × 10^−5^**	1.04 × 10^−4^
	SD	4.09 × 10^−3^	6.03 × 10^−3^	**4.34 × 10^−5^**	4.70 × 10^−4^
f9	Min	6.69 × 10^−3^	**2.36 × 10^−3^**	3.23 × 10^−1^	1.38 × 10^−2^
	Mean	4.05 × 10^0^	4.54 × 10^0^	**2.29 × 10^0^**	2.35 × 10^0^
	SD	3.49 × 10^0^	5.47 × 10^0^	**1.14 × 10^0^**	3.27 × 10^0^
f10	Min	**−5.00 × 10^1^**	−5.00 × 10^1^	−5.00 × 10^1^	−5.00 × 10^1^
	Mean	−4.97 × 10^1^	−4.92 × 10^1^	**−4.98 × 10^1^**	−4.97 × 10^1^
	SD	4.61 × 10^−1^	1.98 × 10^0^	**7.11 × 10^−2^**	1.08 × 10^0^
f11	Min	**−2.10 × 10^2^**	−2.10 × 10^2^	−2.10 × 10^2^	−2.10 × 10^2^
	Mean	−1.45 × 10^2^	−1.46 × 10^2^	**−2.09 × 10^2^**	−2.08 × 10^2^
	SD	7.42 × 10^1^	1.04 × 10^2^	**3.31 × 10^−1^**	6.90 × 10^0^
f12	Min	**6.09 × 10^−4^**	1.15 × 10^−3^	1.09 × 10^0^	4.50 × 10^−1^
	Mean	**3.76 × 10^−1^**	7.09 × 10^−1^	3.26 × 10^0^	8.87 × 10^0^
	SD	**1.05 × 10^0^**	1.35 × 10^0^	5.97 × 10^0^	1.11 × 10^1^
f13	Min	7.43 × 10^−3^	**4.92 × 10^−3^**	1.83 × 10^2^	1.79 × 10^2^
	Mean	**6.38 × 10^−2^**	7.88 × 10^−2^	3.08 × 10^2^	1.24 × 10^3^
	SD	**5.58 × 10^−2^**	8.27 × 10^−2^	7.13 × 10^1^	1.00 × 10^3^
f14	Min	**4.46 × 10^−6^**	4.62 × 10^−6^	1.40 × 10^1^	2.63 × 10^1^
	Mean	**5.38 × 10^−4^**	6.07 × 10^−4^	1.59 × 10^1^	5.74 × 10^8^
	SD	**2.43 × 10^−3^**	3.47 × 10^−3^	8.54 × 10^−1^	2.14 × 10^9^
f15	Min	3.99 × 10^2^	7.38 × 10^2^	**2.66 × 10^1^**	8.80 × 10^2^
	Mean	1.82 × 10^3^	3.28 × 10^3^	**3.67 × 10^1^**	8.24 × 10^3^
	SD	9.73 × 10^2^	1.81 × 10^3^	**7.23 × 10^0^**	2.21 × 10^4^
f16	Min	**3.02 × 10^0^**	5.55 × 10^0^	1.96 × 10^3^	8.25 × 10^4^
	Mean	**1.07 × 10^2^**	1.28 × 10^2^	2.89 × 10^3^	1.64 × 10^6^
	SD	**9.50 × 10^1^**	1.21 × 10^2^	6.14 × 10^2^	1.67 × 10^6^
f17	Min	4.56 × 10^−1^	**4.27 × 10^−2^**	4.07 × 10^2^	8.59 × 10^3^
	Mean	**2.26 × 10^0^**	2.81 × 10^0^	9.65 × 10^2^	1.30 × 10^5^
	SD	**1.44 × 10^0^**	1.88 × 10^0^	2.50 × 10^2^	1.50 × 10^5^
f18	Min	**9.98 × 10^−1^**	9.98 × 10^−1^	9.98 × 10^−1^	9.98 × 10^−1^
	Mean	5.39 × 10^0^	6.12 × 10^0^	**3.82 × 10^0^**	4.17 × 10^0^
	SD	4.47 × 10^0^	5.42 × 10^0^	**2.78 × 10^0^**	3.66 × 10^0^
f19	Min	**3.98 × 10^−1^**	3.98 × 10^−1^	3.98 × 10^−1^	3.98 × 10^−1^
	Mean	**3.98 × 10^−1^**	3.99 × 10^−1^	3.99 × 10^−1^	4.02 × 10^−1^
	SD	**5.55 × 10^−4^**	6.86 × 10^−3^	1.13 × 10^−3^	1.30 × 10^−2^
f20	Min	**1.78 × 10^−10^**	2.70 × 10^−10^	5.41 × 10^−4^	2.03 × 10^−6^
	Mean	2.71 × 10^−2^	3.98 × 10^−2^	1.52 × 10^−2^	**5.47 × 10^−3^**
	SD	1.06 × 10^−1^	1.26 × 10^−1^	1.70 × 10^−2^	**1.08 × 10^−2^**
f21	Min	**1.11 × 10^−10^**	9.75 × 10^−10^	1.77 × 10^−5^	3.06 × 10^−7^
	Mean	**1.10 × 10^−4^**	1.51 × 10^−3^	1.79 × 10^−3^	2.38 × 10^−3^
	SD	**4.42 × 10^−4^**	1.38 × 10^−2^	2.05 × 10^−3^	1.03 × 10^−2^
f22	Min	**2.19 × 10^1^**	2.59 × 10^1^	1.77 × 10^2^	2.09 × 10^2^
	Mean	**5.24 × 10^1^**	5.37 × 10^1^	2.25 × 10^2^	3.20 × 10^2^
	SD	1.52 × 10^1^	**1.38 × 10^1^**	1.81 × 10^1^	4.07 × 10^1^
f23	Min	**−1.26 × 10^4^**	−1.15 × 10^4^	−8.93 × 10^3^	−8.85 × 10^3^
	Mean	**−1.23 × 10^4^**	−1.02 × 10^4^	−6.75 × 10^3^	−7.10 × 10^3^
	SD	**4.71 × 10^2^**	4.85 × 10^2^	9.73 × 10^2^	6.08 × 10^2^
f24	Min	**−1.80 × 10^0^**	−1.80 × 10^0^	−1.80 × 10^0^	−1.80 × 10^0^
	Mean	**−1.80 × 10^0^**	−1.79 × 10^0^	−1.78 × 10^0^	−1.79 × 10^0^
	SD	**1.83 × 10^−6^**	8.26 × 10^−2^	2.57 × 10^−2^	1.13 × 10^−2^
f25	Min	**−4.69 × 10^0^**	−4.69 × 10^0^	−3.92 × 10^0^	−4.37 × 10^0^
	Mean	**−4.53 × 10^0^**	−4.51 × 10^0^	−3.12 × 10^0^	−3.79 × 10^0^
	SD	**1.54 × 10^−1^**	1.69 × 10^−1^	2.70 × 10^−1^	2.50 × 10^−1^
f26	Min	**−9.62 × 10^0^**	−9.61 × 10^0^	−5.92 × 10^0^	−6.32 × 10^0^
	Mean	**−8.95 × 10^0^**	−8.90 × 10^0^	−4.40 × 10^0^	−5.35 × 10^0^
	SD	3.75 × 10^−1^	**3.24 × 10^−1^**	5.02 × 10^−1^	4.84 × 10^−1^
f27	Min	1.77 × 10^−9^	5.70 × 10^−9^	6.23 × 10^−10^	**7.23 × 10^−11^**
	Mean	1.42 × 10^−2^	1.48 × 10^−2^	**1.26 × 10^−6^**	2.75 × 10^−4^
	SD	2.32 × 10^−2^	2.34 × 10^−2^	**1.74 × 10^−6^**	1.39 × 10^−3^
f28	Min	**−1.03 × 10^0^**	−1.03 × 10^0^	−1.03 × 10^0^	−1.03 × 10^0^
	Mean	**−1.02 × 10^0^**	−1.02 × 10^0^	−1.03 × 10^0^	−1.03 × 10^0^
	SD	8.16 × 10^−2^	8.16 × 10^−2^	2.46 × 10^−3^	**1.58 × 10^−3^**
f29	Min	**9.07 × 10^−11^**	5.07 × 10^−10^	1.51 × 10^−4^	9.11 × 10^−6^
	Mean	6.92 × 10^−2^	7.02 × 10^−2^	1.64 × 10^−2^	**3.10 × 10^−3^**
	SD	1.13 × 10^−1^	1.02 × 10^−1^	1.85 × 10^−2^	**6.58 × 10^−3^**
f30	Min	**2.21 × 10^−7^**	4.22 × 10^−7^	1.61 × 10^−4^	8.03 × 10^−6^
	Mean	2.01 × 10^−2^	2.68 × 10^−2^	**5.68 × 10^−3^**	6.61 × 10^−3^
	SD	5.14 × 10^−2^	6.36 × 10^−2^	**6.08 × 10^−3^**	4.05 × 10^−2^
f31	Min	**−1.87 × 10^2^**	−1.87 × 10^2^	−1.87 × 10^2^	−1.87 × 10^2^
	Mean	−1.85 × 10^2^	−1.84 × 10^2^	**−1.86 × 10^2^**	−1.86 × 10^2^
	SD	8.95 × 10^0^	1.24 × 10^1^	**8.93 × 10^−1^**	2.50 × 10^0^
f32	Min	**3.00 × 10^0^**	3.00 × 10^0^	3.00 × 10^0^	3.00 × 10^0^
	Mean	9.63 × 10^0^	1.59 × 10^1^	**3.05 × 10^0^**	3.72 × 10^0^
	SD	1.77 × 10^1^	2.63 × 10^1^	**7.37 × 10^−2^**	3.84 × 10^0^
f33	Min	**3.18 × 10^−4^**	3.42 × 10^−4^	7.12 × 10^−4^	7.16 × 10^−4^
	Mean	2.38 × 10^−3^	6.75 × 10^−3^	4.34 × 10^−3^	**2.13 × 10^−3^**
	SD	4.65 × 10^−3^	8.67 × 10^−3^	7.08 × 10^−3^	**1.22 × 10^−3^**
f34	Min	**−1.02 × 10^1^**	−1.02 × 10^1^	−1.01 × 10^1^	−9.86 × 10^0^
	Mean	**−7.09 × 10^0^**	−5.82 × 10^0^	−5.82 × 10^0^	−5.29 × 10^0^
	SD	2.65 × 10^0^	3.44 × 10^0^	**2.61 × 10^0^**	2.91 × 10^0^
f35	Min	**−1.04 × 10^1^**	−1.04 × 10^1^	−9.41 × 10^0^	−1.03 × 10^1^
	Mean	**−7.41 × 10^0^**	−5.12 × 10^0^	−6.21 × 10^0^	−6.23 × 10^0^
	SD	2.98 × 10^0^	3.18 × 10^0^	**2.29 × 10^0^**	3.23 × 10^0^
f36	Min	**−1.05 × 10^1^**	−1.05 × 10^1^	−1.04 × 10^1^	−1.04 × 10^1^
	Mean	−6.88 × 10^0^	−5.85 × 10^0^	−6.85 × 10^0^	**−6.97 × 10^0^**
	SD	3.25 × 10^0^	3.58 × 10^0^	**2.38 × 10^0^**	2.96 × 10^0^
f37	Min	5.99 × 10^−3^	1.51 × 10^−2^	4.83 × 10^−2^	**5.32 × 10^−3^**
	Mean	**4.01 × 10^0^**	1.24 × 10^1^	3.68 × 10^1^	4.16 × 10^0^
	SD	**6.90 × 10^0^**	1.43 × 10^1^	1.77 × 10^2^	1.66 × 10^1^
f38	Min	4.69 × 10^−4^	**3.27 × 10^−4^**	3.33 × 10^−2^	8.12 × 10^−3^
	Mean	**1.13 × 10^−1^**	1.68 × 10^−1^	9.39 × 10^−1^	1.71 × 10^−1^
	SD	**2.39 × 10^−1^**	3.83 × 10^−1^	2.90 × 10^0^	2.56 × 10^−1^
f39	Min	**−3.86 × 10^0^**	−3.86 × 10^0^	−3.86 × 10^0^	−3.86 × 10^0^
	Mean	**−3.85 × 10^0^**	−3.82 × 10^0^	−3.82 × 10^0^	−3.80 × 10^0^
	SD	1.09 × 10^−1^	1.85 × 10^−1^	2.50 × 10^−1^	**5.11 × 10^−2^**
f40	Min	**−3.32 × 10^0^**	−3.32 × 10^0^	−3.21 × 10^0^	−3.10 × 10^0^
	Mean	**−3.28 × 10^0^**	−3.27 × 10^0^	−2.82 × 10^0^	−2.25 × 10^0^
	SD	**5.70 × 10^−2^**	5.90 × 10^−2^	4.60 × 10^−1^	5.13 × 10^−1^
f41	Min	4.80 × 10^−7^	**2.73 × 10^−7^**	2.81 × 10^−1^	9.71 × 10^−1^
	Mean	**7.31 × 10^−2^**	1.03 × 10^−1^	5.08 × 10^−1^	1.12 × 10^0^
	SD	1.11 × 10^−1^	2.78 × 10^−1^	**5.85 × 10^−2^**	2.35 × 10^−1^
f42	Min	**1.21 × 10^−4^**	1.16 × 10^0^	3.81 × 10^0^	6.73 × 10^0^
	Mean	**2.68 × 10^0^**	3.15 × 10^0^	4.17 × 10^0^	1.59 × 10^1^
	SD	1.04 × 10^0^	1.32 × 10^0^	**1.40 × 10^−1^**	4.55 × 10^0^
f43	Min	1.75 × 10^−7^	**4.08 × 10^−8^**	2.37 × 10^−1^	8.06 × 10^1^
	Mean	**3.51 × 10^−1^**	5.15 × 10^−1^	2.24 × 10^0^	6.38 × 10^5^
	SD	**7.25 × 10^−1^**	8.52 × 10^−1^	3.17 × 10^0^	2.76 × 10^6^
f44	Min	6.65 × 10^−5^	**1.82 × 10^−5^**	1.64 × 10^0^	1.40 × 10^3^
	Mean	**1.32 × 10^0^**	2.06 × 10^0^	2.13 × 10^0^	8.28 × 10^5^
	SD	2.49 × 10^0^	4.04 × 10^0^	**2.12 × 10^−1^**	3.11 × 10^6^
f45	Min	**−1.08 × 10^0^**	−1.08 × 10^0^	−1.08 × 10^0^	−1.08 × 10^0^
	Mean	−1.02 × 10^0^	−9.84 × 10^−1^	**−1.07 × 10^0^**	−1.03 × 10^0^
	SD	1.05 × 10^−1^	1.65 × 10^−1^	**2.28 × 10^−2^**	8.82 × 10^−2^
f46	Min	**−1.50 × 10^0^**	−1.50 × 10^0^	−1.49 × 10^0^	−1.50 × 10^0^
	Mean	−8.85 × 10^−1^	−7.46 × 10^−1^	−9.35 × 10^−1^	**−9.52 × 10^−1^**
	SD	3.18 × 10^−1^	**2.47 × 10^−1^**	2.60 × 10^−1^	3.86 × 10^−1^
f47	Min	**−7.98 × 10^−1^**	−7.98 × 10^−1^	−7.98 × 10^−1^	−7.98 × 10^−1^
	Mean	**−4.99 × 10^−1^**	−4.51 × 10^−1^	−4.72 × 10^−1^	−2.20 × 10^−1^
	SD	2.25 × 10^−1^	**2.09 × 10^−1^**	2.12 × 10^−1^	2.11 × 10^−1^
f48	Min	7.98 × 10^−11^	**5.08 × 10^−11^**	1.68 × 10^−2^	3.32 × 10^−4^
	Mean	**1.32 × 10^−5^**	2.37 × 10^−4^	9.97 × 10^1^	3.71 × 10^1^
	SD	**3.41 × 10^−5^**	2.22 × 10^−3^	2.47 × 10^2^	1.70 × 10^2^
f49	Min	6.66 × 10^−4^	**5.12 × 10^−5^**	1.01 × 10^2^	3.18 × 10^1^
	Mean	**3.09 × 10^2^**	5.31 × 10^2^	9.98 × 10^2^	1.95 × 10^3^
	SD	**6.69 × 10^2^**	8.78 × 10^2^	1.26 × 10^3^	1.84 × 10^3^
f50	Min	5.87 × 10^−1^	**1.68 × 10^−1^**	3.01 × 10^3^	1.40 × 10^3^
	Mean	**1.26 × 10^3^**	3.16 × 10^3^	1.22 × 10^4^	3.85 × 10^4^
	SD	**2.26 × 10^3^**	4.72 × 10^3^	9.65 × 10^3^	2.39 × 10^4^

**Table 4:** Friedman test statistical results of four algorithms on 50 test functions (f1~f50).

Source	SS	df	MS	Chi-sq	Prob > Chi-sq
Columns	116.13	3	38.71	77.13	1.26353 × 10^−16^
Error	561.37	447	1.2559		
Total	677.5	599			

**Table 5:** The UAV position, target position, the stereoscopic threat distribution field position, and the effective operating distance in the four independent comparative experimental scenarios.

Case Number	UAV Position	Target Position	Stereoscopic Threat Distribution Field Position	Effective Operating Distance
1	(0,60,0) (0,60,0) (0,60,0) (0,60,0)	(85,80,100) (85,80,100) (85,80,100) (85,80,100)	(60,70,45) (20,60,10) (70,65,90) (60,80,70) (35,70,30) (60,50,80)	10 8 3 8 8 5
2	(0,72,0) (0,72,0) (0,72,0) (0,72,0) (0,72,0) (0,72,0) (0,72,0) (0,72,0)	(85,55,100) (85,60,100) (85,65,100) (85,70,100) (85,75,100) (85,80,100) (85,85,100) (85,90,100)	(60,70,45) (20,60,10) (70,65,90) (35,70,30) (60,50,80)	10 8 3 8 5
3	(0,25,0) (0,35,0) (0,45,0) (0,55,0) (0,65,0) (0,75,0) (0,85,0) (0,95,0)	(85,80,100) (85,80,100) (85,80,100) (85,80,100) (85,80,100) (85,80,100) (85,80,100) (85,80,100)	(60,70,45) (20,60,10) (70,65,90) (60,80,70) (35,70,30) (60,50,80)	10 8 3 8 8 5
4	(0,25,0) (0,35,0) (0,45,0) (0,55,0) (0,65,0) (0,75,0) (0,85,0) (0,95,0)	(85,55,100) (85,60,100) (85,65,100) (85,70,100) (85,75,100) (85,80,100) (85,85,100) (85,90,100)	(60,70,45) (20,60,10) (70,65,90) (60,80,70) (35,70,30) (60,50,80)	10 8 3 8 8 5

**Table 6:** Comparison of the extremes, means, and variances of 50 simulations in four scenarios.

Functions	Properties	MCO	CO	PSO	GWO
Case1	Min	**1.81 × 10^4^**	1.81 × 10^4^	2.07 × 10^4^	1.81 × 10^4^
	Mean	**1.82 × 10^4^**	1.82 × 10^4^	2.09 × 10^4^	1.82 × 10^4^
	SD	**5.16 × 10^1^**	2.05 × 10^2^	1.38 × 10^2^	2.39 × 10^2^
Case2	Min	**3.54 × 10^4^**	3.54 × 10^4^	4.16 × 10^4^	3.60 × 10^4^
	Mean	**3.56 × 10^4^**	3.57 × 10^4^	8.00 × 10^9^	3.64 × 10^4^
	SD	**1.58 × 10^2^**	2.03 × 10^2^	4.22 × 10^9^	4.60 × 10^2^
Case3	Min	**3.48 × 10^4^**	3.73 × 10^4^	3.82 × 10^4^	3.68 × 10^4^
	Mean	**3.49 × 10^4^**	3.89 × 10^4^	3.91 × 10^4^	3.75 × 10^4^
	SD	**4.6 × 10^2^**	6.15 × 10^2^	5.32 × 10^2^	3.39 × 10^2^
Case4	Min	**3.47 × 10^4^**	3.49 × 10^4^	3.88 × 10^4^	3.54 × 10^4^
	Mean	**3.49 × 10^4^**	3.51 × 10^4^	3.96 × 10^4^	3.58 × 10^4^
	SD	**1.74 × 10^2^**	1.74 × 10^2^	5.05 × 10^2^	2.74 × 10^2^

## Data Availability

The data that support the findings of this study are available from the corresponding author upon reasonable request.
